# Quantitative comparison of a novel wide-field OCT-angiography device with ultrawide-field fluorescein angiography in detecting retinal nonperfusion in vascular retinopathies

**DOI:** 10.1186/s12886-025-04468-z

**Published:** 2025-11-13

**Authors:** Michael Hafner, Tina R. Herold, Viktoria Deiters, Bettina von Livonius, Siegfried G. Priglinger, Maximilian J. Gerhardt

**Affiliations:** Department of Ophthalmology, LMU University Hospital, Ludwig-Maximilians-Universität München, Mathildenstraße 8, 80336 Munich, Germany

**Keywords:** Wide-field OCTA, Retinal nonperfusion, Diabetic retinopathy (DR), Retinal vein occlusion (RVO), Ischemic index (ISI), Swept-source OCTA, Semi-automated segmentation, DREAM OCT

## Abstract

**Background:**

Reliable assessment of retinal nonperfusion is critical in managing vascular retinopathies. While ultrawide-field fluorescein angiography (UWF-FA) is the clinical standard, it is invasive and dye-dependent. Previous wide-field optical coherence tomography angiography (WF-OCTA) systems have been limited by insufficient peripheral coverage. To the best of our knowledge, this is the first quantitative comparison of DREAM WF-OCTA with UWF-FA. The study leverages the device’s increased field of view (≈130° single scan, > 200° montage) and demonstrates that the previously published, semi-automated VMseg approach can also be applied to DREAM data.

**Methods:**

24 eyes from 13 patients with diabetic retinopathy or retinal vein occlusion underwent both UWF-FA (Optos Silverstone, 200°) and WF-OCTA and were analyzed. The ischemic index (ISI) was calculated for each modality using previously developed semi-automated segmentation (VMseg) for WF-OCTA and manual annotation for UWF-FA. Agreement was assessed using correlation, linear regression, and Bland-Altman analyses.

**Results:**

ISI values from WF-OCTA showed strong correlations with UWF-FA (*r* = 0.92 for central, *r* = 0.96 for montage). Central WF-OCTA showed good absolute agreement with UWF-FA in mild ischemia, montage WF-OCTA with extended coverage in mild to moderate and partly severe ischemia. However, Bland-Altman analysis revealed proportional bias with increasing underestimation at higher nonperfusion levels, indicating that field of view limitations persist despite technological advances.

**Conclusions:**

This study demonstrates that the DREAM OCT™ system with improved peripheral coverage enables reliable non-invasive assessment of retinal ischemia in mild to moderate cases. However, WF-OCTA should be considered complementary to UWF-FA, particularly in severe peripheral ischemia. Semi-automated segmentation enhances reproducibility and supports broader clinical adoption of OCTA in ischemia monitoring.

**Trial registration:**

Ethics approval was granted by the Institutional Review Board of the Faculty of Medicine at LMU Munich (study ID: 24–0571), and the study was conducted following the principles of the Declaration of Helsinki. All participants provided written informed consent before inclusion in the study.

## Introduction

Since its introduction, optical coherence tomography angiography (OCTA) has rapidly become an essential diagnostic tool for chorioretinal and vascular diseases [1]. In contrast to traditional methods such as fluorescein angiography (FA) and indocyanine green angiography (ICGA), OCTA is non-invasive and does not require intravenous dye injections. Therefore, there is no risk of allergic reactions, it is more comfortable for the patients and expands suitability for a broader patient population [2]. Further advantages of OCTA are fast image acquisition, high-resolution imaging of capillaries without interference from dye leakage, and the capacity for depth-resolved analysis, which enables detailed evaluation of blood flow in specific layers of the retina and choroid [3].

However, the clinical need for wider fields of view in OCTA has been recognized for several years. Previous investigations have demonstrated that standard OCTA devices, typically offering fields of view ranging from 3 × 3 mm to 15 × 15 mm, provide insufficient coverage for comprehensive assessment of vascular retinopathies, particularly in detecting peripheral ischemia [4, 5]. Mosaic techniques have been developed to address this limitation, but these approaches are time-consuming, prone to artefacts, and not universally available in clinical practice [6].

Recently, Intalight Inc. (San Jose, California, USA) launched a new Swept-Source OCTA device, the DREAM OCT™ [7], designed to address the recognized need for wider OCTA coverage. This system facilitates wide-field OCTA (WF-OCTA) imaging, capturing a 26 mm × 21 mm field of view (inner angle of 130°) with a single scan [8], representing a significant advancement over previously utilized devices such as the Zeiss Plex® Elite 9000 (15 × 15 mm) or the Canon Xephilio OCT-S1 (23 × 20 mm) [6]. Additionally, DREAM OCT provides a montage feature that, together with optical design, enables a field of view potentially over 200°, approaching – but not matching – the spatial coverage of UWF-FA while maintaining the non-invasive advantages of OCTA [8]. However, whether this technological advancement fully addresses the clinical limitations identified in previous WF-OCTA studies remains to be systematically evaluated.

This study aims to quantitatively compare this novel WF-OCTA device, the DREAM OCT, with UWF-FA in patients with retinal nonperfusion due to diabetic retinopathy or retinal vein occlusion. The primary objective was to assess differences in the detection of nonperfusion areas between modalities. To our knowledge, this is the first study to quantitatively compare DREAM WF-OCTA with UWF-FA in patients with retinal vascular disease.

## Methods

Ethics approval was granted by the Institutional Review Board of the Faculty of Medicine at LMU Munich (study ID: 24–0571), and the study was conducted following the principles of the Declaration of Helsinki. All participants provided informed consent before inclusion in the study. The imaging data included WF-OCTA images acquired with the Intalight DREAM OCT system (model VG200D, central image, and montage image, the latter consisting of five merged single scans), alongside UWF-FA images captured with the Optos Silverstone device (Optos plc, Dunfermline, Scotland, United Kingdom).

### Participants

Patients were recruited from the Department of Ophthalmology at LMU University Hospital Munich between December 2024 and February 2025. The inclusion criteria were as follows: (i) age 18 years or older, (ii) presence of retinal nonperfusion area resulting from diabetic retinopathy or retinal vein occlusion, (iii) absence of confounding ocular conditions, such as intraocular infections or uveitis, (iv) clear optic media (e.g., no cataract, no vitreous bleeding), (v) no contraindication for FA (e.g., allergy to fluorescein).

### Imaging

**Fig. 1:**
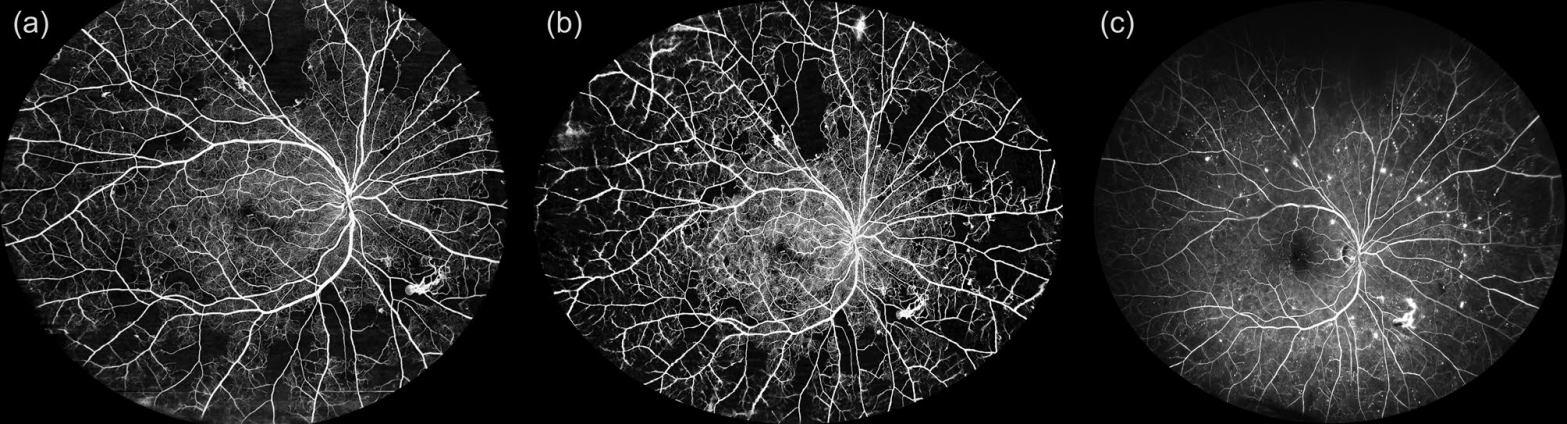
Comparison of retinal nonperfusion across central and montage WF-OCTA and UWF-FA. This figure illustrates retinal nonperfusion in a representative eye using three imaging modalities. (**a**) Central wide-field optical coherence tomography angiography (WF-OCTA) image centered on the fovea, acquired with a 26 mm × 21 mm single scan using the DREAM OCT™ system. (**b**) Montage WF-OCTA image combining five peripheral scans into a single wide-field view, providing improved visualization of peripheral ischemic areas. (**c**) Ultrawide-field fluorescein angiography (UWF-FA) image acquired with the Optos Silverstone system, displaying retinal nonperfusion across the posterior pole and peripheral retina. The figure demonstrates the differences in field of view and vascular detail between imaging modalities. While UWF-FA provides broader peripheral coverage, WF-OCTA offers higher-resolution visualization of microvascular details and more precise delineation of ischemic regions in the central and mid-peripheral retina

**Fig. 2:**
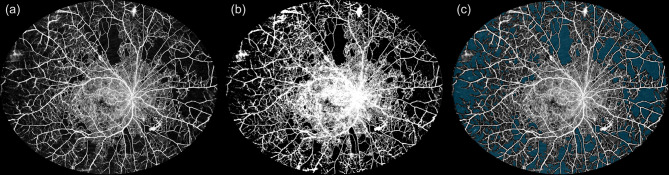
Image processing for quantitative analysis of WF-OCTA images. This figure demonstrates the image processing steps used to quantify vessel density and ischemic index (ISI) in wide-field OCTA (WF-OCTA) images. (**a**) Original montage WF-OCTA image acquired with DREAM OCT™ showing retinal vasculature and nonperfusion areas. (**b**) Binarized image generated using Huang thresholding in Fiji to calculate vessel density (VD). White pixels represent perfused vasculature, while black pixels indicate nonperfused areas. The vessel density-derived ischemic index (VD-ISI) was calculated as 1 – VD. (**c**) Montage WF-OCTA image with nonperfusion areas semi-automatically segmented using VMseg, a variance-map-based segmentation algorithm. This step enables standardized and semi-automated isi computation

WF-OCTA and UWF-FA images were captured on the same day. Pupillary dilation was performed beforehand to ensure optimal image quality. For central WF-OCTA imaging, single scans covering 26 mm × 21 mm centered on the fovea were acquired. For montage WF-OCTA, five separate 26 mm × 21 mm images were captured, covering the central, nasal-superior, nasal-inferior, temporal-superior, and temporal-inferior quadrants. These images were subsequently merged into a single montage image using the proprietary Intalight DREAM OCT software. The DREAM OCT system provides an axial resolution of ≤ 5.5 µm and a transverse resolution of ≤ 15 µm in tissue. To improve image quality and reduce artefacts, it employs the proprietary TRUE Angio™ algorithm (Trans-dimensional Reconstructed Ultra-sensitive Enhanced Angio Algorithm), which enhances blood flow sensitivity and reduces projection artefacts. This is combined with Deep Layer™ segmentation technology for improved vascular layer distinction and artefact suppression [9]. All WF-OCTA scans were evaluated for signal strength and artefacts prior to analysis. A signal strength index ≥ 7 (DREAM scale) was required for inclusion. Scans with significant motion artefacts or media opacities (e.g., cataract) were excluded. UWF-FA images were manually inspected for overall contrast and vessel delineation. Examples of the resulting images are shown in Fig. 1 (a: central WF-OCTA, b: montage WF-OCTA, c: UWF-FA) and Fig. 2a (montage WF-OCTA).

### Image processing and analysis

As a first step, regions of WF-OCTA and UWF-FA images affected by imaging artefacts (e.g., shadowing or low signal) were manually cropped prior to further analysis.

For WF-OCTA analysis, vessel density (VD) and ischemic index (ISI) were used as quantitative parameters. To determine VD, WF-OCTA images were binarized in Fiji (National Institutes of Health, MD, USA) using Huang thresholding on 8-bit images (an example of a binarized image is shown in Fig. 2b). The perfused area was quantified by dividing the white pixel count (representing perfused regions) by the total pixel count, thereby reflecting the vascular area ratio. To make this more comparable to ISI, which represents an area of retinal nonperfusion, a vessel-density derived ischemic index (VD-ISI) was calculated using (VD-ISI) = 1 - VD. This indicates the avascular area ratio, represented by the black pixel count (non-vascular regions) to the total pixel count.

Although binarization methods such as Huang thresholding have primarily been validated in smaller OCTA scan formats, we applied this approach to wide-field datasets to provide a simple global estimate of perfused area. However, recognizing the limitations of such threshold-based methods in large-field scans, particularly due to increased heterogeneity and susceptibility to artefacts, we additionally employed the semi-automated VMseg algorithm, which was developed to address these shortcomings by incorporating local structural variance rather than relying solely on intensity-based thresholding [10]. By identifying nonperfused areas through VMseg, which was previously developed by Le Boité et al. [10], we further calculated ISI in WF-OCTA images similar to UWF-FA. VMseg binarizes a variance map produced through convolution and morphological operations. To suppress segmentation noise and exclude clinically irrelevant microlesions, we applied a minimum area threshold of 500 pixels, corresponding to approximately 0.15 mm^2^ at the given image resolution of the DREAM system. This is consistent with the threshold defined in the original VMseg publication of 0.15 mm^2^ [10], adapted for the different field of view and resolution of the DREAM system. However, due to peripheral image distortion and local scaling inaccuracies, particularly toward the image edges, direct metric conversion may be imprecise. For segmentation of nonperfusion areas using VMseg, we applied the optimal parameters as reported in the original VMseg publication [10]: intensity threshold = 75, variance threshold = 17, number of morphological iterations = 1, kernel size combination = 3,5, and α (variance regularization factor) = 1. Nonperfusion size threshold was adapted to 500 pixels, consistent with the original publication and adapted to DREAM’s resolution.

To assess the applicability of VMseg to DREAM WF-OCTA images, we conducted a validation substudy using ten representative scans (randomly selected; six with diabetic retinopathy and four with retinal vein occlusion). Manually segmented nonperfusion areas (created by placing region-of-interest annotations using Fiji software) were compared with VMseg output, and agreement was quantified using the Dice-Sørensen coefficient [11, 12]. Dice values were computed in Python using NumPy-based binary mask comparison. An example of a montage WF-OCTA image annotated by VMseg is provided in Fig. 2c.

**Fig. 3:**
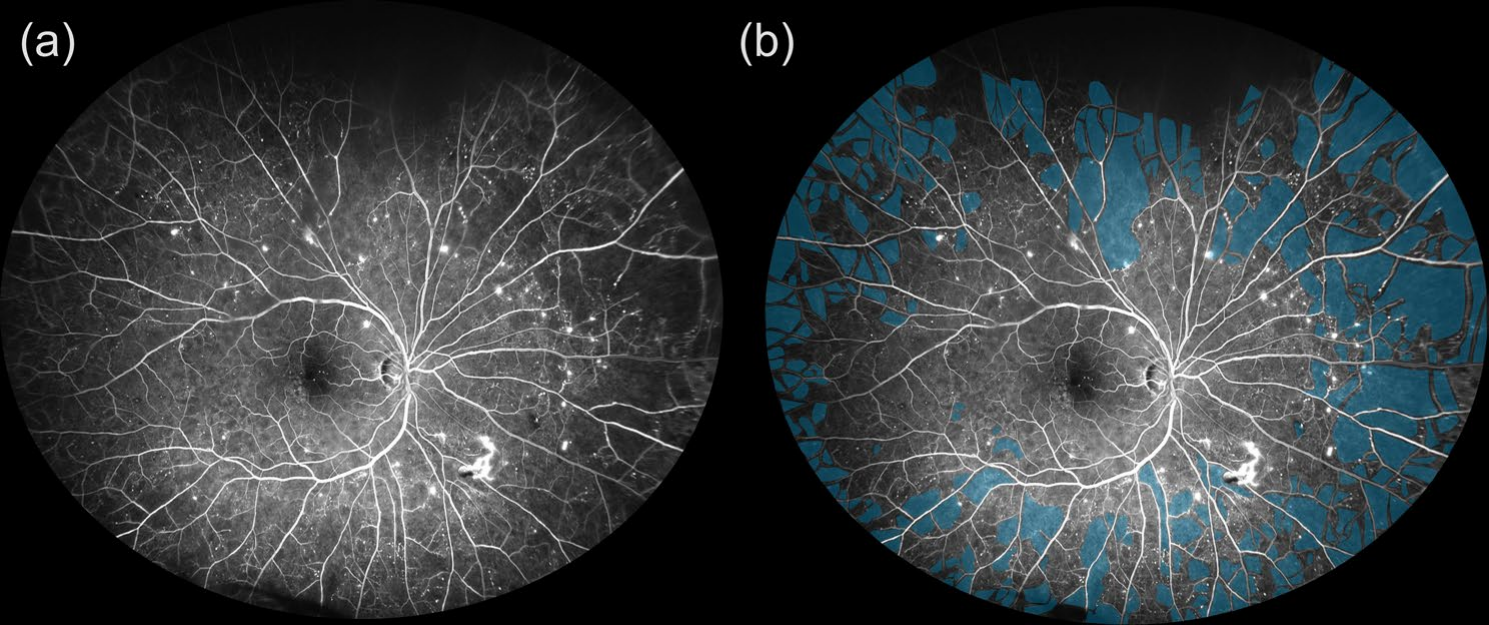
Manual segmentation of nonperfusion areas in UWF-FA for isi calculation. (**a**) Unannotated original ultrawide-field fluorescein angiography (UWF-FA) image showing peripheral capillary dropout in a patient with diabetic retinopathy. (**b**) Manual segmentation of nonperfused retinal areas (blue) using Fiji software. Regions of interest were drawn to delineate ischemic zones based on capillary loss and hypofluorescence. The ischemic index (ISI) was computed as the proportion of nonperfused area relative to the total analyzable retinal area

In UWF-FA imaging, nonperfusion areas were manually segmented by placing region-of-interest annotations using Fiji software. All segmentations were performed by a single experienced grader (M.H.) who was masked to the OCTA results to avoid bias. This approach ensured consistent interpretation across the dataset and minimized variability in region definition. The ISI was calculated by dividing the total nonperfused area by the entire image area, which includes both perfused and nonperfused regions. This approach uses established area ratio calculation to address mapping distortion [13, 14]. An annotated example of a UWF-FA image (unannotated image in Fig. 3a) illustrating this method is shown in Fig. 3b.

For UWF-FA, nonperfusion areas were defined as well-demarcated zones without capillary filling, excluding regions of blocked fluorescence (e.g., hemorrhage or media opacities). For WF-OCTA, nonperfusion areas were defined as contiguous areas lacking vascular signal in the absence of motion artefacts, projection artefacts, or shadowing. For improved interpretability, ischemia was classified according to ISI thresholds as follows: mild (≤20%), moderate (21–40%), and severe ( > 40%) ischemia.

### Data analysis and statistics

Data management was conducted in Microsoft Excel (Version 16.78.3 for Mac), with statistical analysis performed using GraphPad Prism (Version 10.3.1 for macOS). A significant threshold of *p* < 0.05 was applied for all tests. Due to non-normality, as indicated by the Shapiro-Wilk test, data is presented as median and interquartile ranges (IQR). Differences in ISI among the groups were evaluated using the Friedman ANOVA test, followed by Dunn’s multiple comparisons test for post-hoc analysis. Additionally, linear regression analysis and Bland-Altman analysis were used to further examine the data. A post-hoc power analysis was conducted using G*Power 3.1.9.6 [15] to assess the sensitivity of the primary correlation analyses.

### Medical writing, editorial, and other assistance

During the preparation of this work, the authors used Grammarly (http://www.grammarly.com) to refine the academic language and grammar of the editorial. After using this tool, the authors reviewed and edited the content as needed and take full responsibility for the content of the publication.

## Results

### Baseline demographics

The participants had an average age of 54.94 ± 11.79 years (mean ± standard deviation), with six women and seven men. Seven patients showed retinal nonperfusion due to diabetic retinopathy, whereas six patients exhibited nonperfusion because of retinal vein occlusion (see Table 1).

**Table 1:** Baseline demographic characteristics and clinical features of study participants

Number of patients	13
Number of eyes	24
Mean age (years)	54.94 ± 11.79
Gender	
Male	7 patients
Female	6 patients
Diagnosis	
Diabetic retinopathy	7 patients
Retinal vein occlusion	6 patients

This table presents the baseline demographic and clinical characteristics of the 13 patients included in the study. The average age and gender distribution are reported. The distribution between diabetic retinopathy and retinal vein occlusion is shown.

A total of 24 eyes were included in the final analysis after exclusion of two eyes due to insufficient WF-OCTA image quality. Of the analyzed eyes, 13 were right and 11 were left eyes. Twelve eyes were diagnosed with diabetic retinopathy, including 2 eyes with moderate non-proliferative diabetic retinopathy, 2 with severe non-proliferative diabetic retinopathy, and 8 with proliferative diabetic retinopathy. The remaining 12 eyes had retinal vein occlusion, predominantly branch retinal vein occlusion (*n* = 9). Three eyes were classified as partner eyes, defined as fellow eyes without clinical RVO diagnosis but with potentially focal nonperfusion areas. Macular edema was present in 1 of the included eyes (see Table 2).

**Table 2:** Eye-level characteristics (*n* = 24 eyes)

Total number of eyes imaged Right eyes Left eyes	26 13 13
Eyes excluded due to image quality in WF-OCTA	2
Total number of eyes analyzed	24
Right eyes	13
Left eyes	11
Diagnosis: DR	12
Mild NPDR Moderate NPDR	0 2
Severe NPDR PDR	2 8
Diagnosis: RVO	12
BRVO	9
CRVO Partner eyes	0 3
Eyes with macular edema	1

This table presents the clinical characteristics of all eyes included in the analysis. Laterality, diagnosis (diabetic retinopathy [DR] vs. retinal vein occlusion [RVO]), DR severity (non-proliferative diabetic retinopathy [NPDR] and proliferative diabetic retinopathy [PDR]), and the presence of macular edema are summarized. Among RVO eyes, the majority had branch retinal vein occlusion (BRVO); no eyes with central retinal vein occlusion (CRVO) were included. “Partner eyes” (fellow eyes without clinically apparent RVO, but partially showing small nonperfusion areas) were also analyzed. Two eyes were excluded due to insufficient image quality in wide-field optical coherence tomography angiography (WF-OCTA).

### VMseg validation and ISI measurements

To confirm the applicability of VMseg to DREAM WF-OCTA data, we compared its segmentation to manual annotations in ten representative images, yielding a mean Dice coefficient of 0.72 ± 0.12 – consistent with values reported for the Zeiss Plex® Elite 9000 [10].

For central WF-OCTA images, ISI was measured at 16.35% (IQR: 25.864%), while montage WF-OCTA images showed a median ISI of 23.77% (IQR: 31.05%). The median VD-ISI for central WF-OCTA was measured at 62.58% (IQR: 6.54%) and 59.83% (IQR: 9.59%) for montage WF-OCTA.

In the case of UWF-FA, ISI was measured at 15.83% (IQR: 37.232%). Pairwise comparisons between ISI value distributions in central or montage WF-OCTA and UWF-FA did not reveal significant differences.

**Fig. 4:**
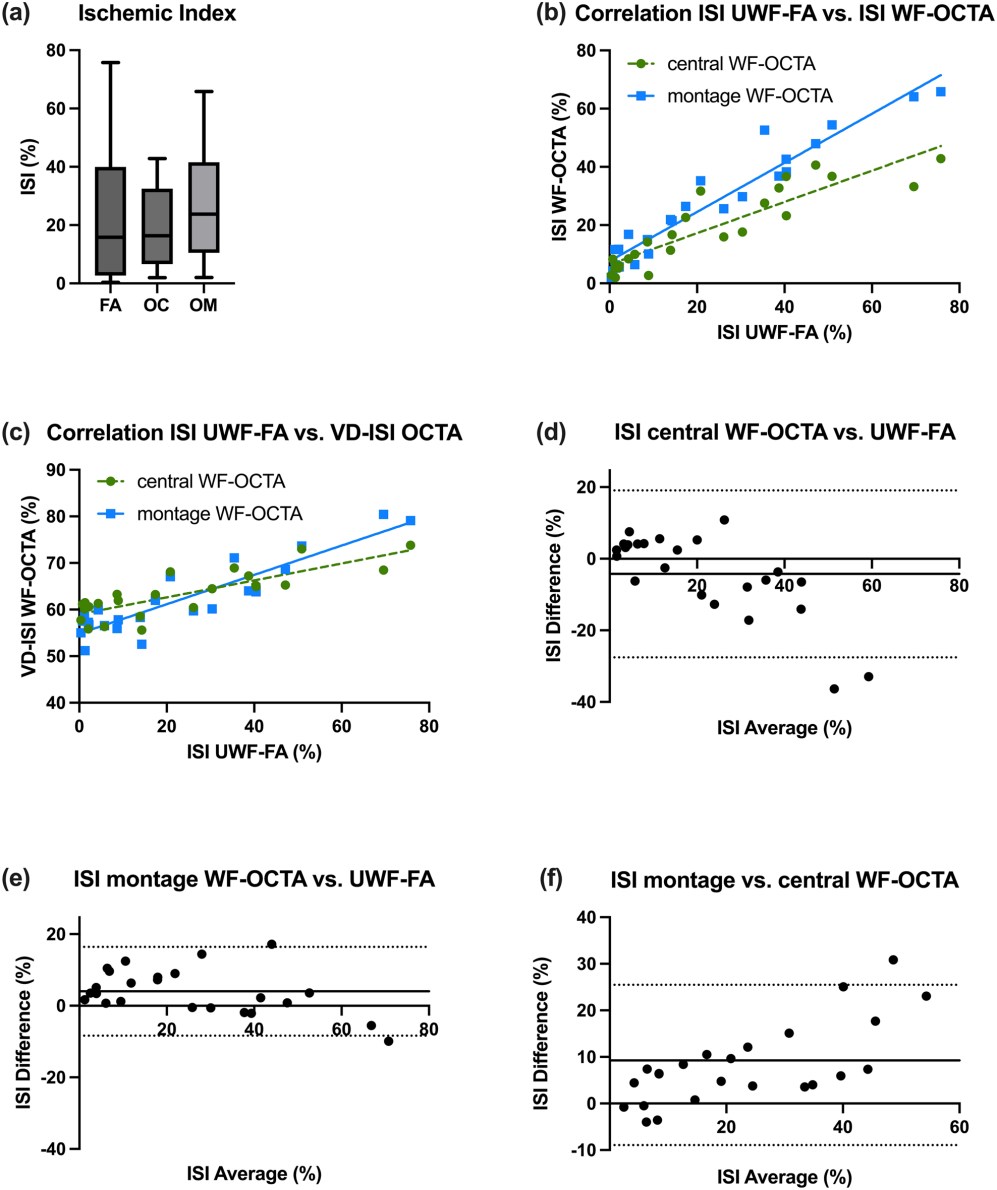
Quantitative comparison of isi across UWF-FA and WF-OCTA using multiple analyses. (**a**) Box plot comparing ISI values from UWF-FA (FA), central WF-OCTA (OC), and montage WF-OCTA (OM). No significant differences were detected (Friedman ANOVA). (**b**) Scatter plot and linear regression showing the correlation between UWF-FA ISI and WF-OCTA isi (central and montage), with a stronger agreement in the montage protocol. (**c**) Correlation between UWF-FA ISI and VD-ISI from central and montage WF-OCTA. (**d**–**f**) Bland-Altman plots comparing: (**d**) Central WF-OCTA vs. UWF-FA ISI. (**e**) Montage WF-OCTA vs. UWF-FA ISI. (**f**) Montage vs. central WF-OCTA ISI. Each plot includes bias (solid line) and 95% limits of agreement (dashed lines)

Table 3 contains detailed data, graphically illustrated in Fig. 4a.

**Table 3:** Quantitative comparison of the ischemic index (ISI) and vessel density-derived isi (VD-ISI) between UWF-FA and WF-OCTA (central and montage scans)

	ISI (UWF-FA)	ISI (central WF-OCTA)	ISI (montage WF-OCTA)	VD-ISI (central WF-OCTA)	VD-ISI (montage WF-OCTA)
median (%)	15.83	16.35	23.77	62.58	59.83
IQR (%)	37.232	25.864	31.05	6.54	9.59
p	-	> 0.9999	> 0.9999	-	-
Spearman´s r	-	0.9209	0.9557	0.7783	0.84
p_Spearman_	-	** < 0.0001**	** < 0.0001**	** < 0.0001**	** < 0.0001**
Regression slope	-	0.5364	0.8432	0.1817	0.3148
Y-intercept (%)	-	6.504	7.658	59.00	54.86
p_regression_	-	** < 0.0001**	** < 0.0001**	** < 0.0001**	** < 0.0001**

This table summarizes the ischemic index (ISI) values and vessel density-derived ISI (VD-ISI) in ultrawide-field fluorescein angiography (UWF-FA) and wide-field OCT angiography (WF-OCTA) using central and montage scan protocols. Median values and interquartile ranges (IQR) are provided, alongside the results of Friedman ANOVA with post-hoc analysis for pairwise comparisons between imaging modalities. Correlation analyses using Spearman’s rank coefficient evaluate the relationship of UWF-FA ISI with OCTA-based parameters. Linear regression results (slope, Y-intercept, and significance level) assess quantitative agreement between modalities. Significant p-values are reported in bold

### Agreement between WF-OCTA and UWF-FA

Correlation analysis evaluated the relationship between WF-OCTA-derived metrics and ISI in UWF-FA (considered the well-established gold standard for comparison [16]). Spearman’s correlation coefficient (r) for ISI in central WF-OCTA was 0.9209 (*p* < 0.0001) and for montage WF-OCTA 0.9557 (*p* < 0.0001). For VD-ISI, Spearman’s r was 0.7783 (*p* < 0.0001) for central WF-OCTA and 0.84 (*p* < 0.0001) for montage WF-OCTA. These coefficients are summarized in Table 3.

While both ISI and VD-ISI aim to reflect retinal perfusion, they are derived from fundamentally different methods: ISI is based on area segmentation of nonperfusion, while VD-ISI reflects global vascular density via pixel binarization. Given these differences, they are not directly comparable in scale. Correlation analysis was used to explore their relationship, but no statistical comparison of absolute values was performed.

### Quantitative relationship

To further investigate the relations between imaging modalities and analysis methods, linear regression analysis was conducted. For the ISI comparison between UWF-FA and central WF-OCTA, a regression slope of 0.5364 with a Y-intercept of 6.504% was identified. In the UWF-FA and montage WF-OCTA comparison, the regression slope was 0.8432 with a Y-intercept of 7.658%. When assessing UWF-FA ISI against VD-ISI in central WF-OCTA, the regression slope was 0.1817 with a Y-intercept of 59.00%, while for montage WF-OCTA, the slope was 0.3148 with a Y-intercept of 54.86%. Each regression analysis exhibited *p* < 0.0001.

Detailed results are displayed in Table 3, and the linear regression models are plotted in Fig. 4b and c.

### Assessment of measurement differences

To better understand the differences between the imaging techniques and analysis methods, a Bland-Altman analysis was conducted to compare the ISI values from UWF-FA (considered the established gold standard) with those from WF-OCTA. Results are given in Table 4.

**Table 4:** Bland-Altman analysis of agreement between ischemic index (isi) measurements from WF-OCTA and UWF-FA

	ISI (central WF-OCTA) vs. ISI (UWF-FA)	ISI (montage WF-OCTA) vs. ISI (UWF-FA)	ISI (montage WF-OCTA) vs. ISI (central WF-OCTA)
Bias (%)	−4.248	4.022	8.269
SD of bias (%)	11.89	6.326	8.78
95% limits of agreement			
From (%)	−27.56	−8.377	−8.939
To (%)	19.06	16.42	25.48

This table presents the results of Bland-Altman analyses comparing ischemic index (ISI) measurements between ultrawide-field fluorescein angiography (UWF-FA) and wide-field OCT angiography (WF-OCTA), using both central and montage scan protocols. The analysis includes the mean bias (average difference), standard deviation (SD) of bias, and the 95% limits of agreement, defined as the range within which 95% of the differences between paired measurements fall. The comparison highlights the level of agreement between imaging modalities and scan types and quantifies potential systematic differences in ISI estimation

Bland-Altman analysis revealed proportional bias between UWF-FA and central WF-OCTA. At low ISI values, central WF-OCTA showed slight overestimation, while progressively underestimating ISI with increasing nonperfusion severity. This proportional bias pattern indicates field-of-view dependent measurement differences rather than constant systematic error. This trend is illustrated in Fig. 4d.

In contrast, Bland-Altman comparison between UWF-FA and montage WF-OCTA revealed closer absolute agreement, particularly for mild to moderate ischemia. Although montage WF-OCTA initially yields slightly higher ISI values in mild ischemia, it demonstrates better absolute agreement with UWF-FA in moderate nonperfusion compared to central WF-OCTA. However, the bias pattern shows that even montage WF-OCTA progressively tends to underestimate ISI with increasingly severe ischemia. This comparison is shown in Fig. 4e.

When comparing the ISI values between central WF-OCTA and montage WF-OCTA images, we observed a good alignment for mild ischemia. However, as the ISI values increased, the agreement diminished, with central WF-OCTA images displaying lower ISI values compared to those obtained from montage WF-OCTA images in moderate to severe ischemia. This trend is illustrated in Fig. 4f.

### Power analysis

For the observed correlation between ISI derived from montage WF-OCTA and UWF-FA (Spearman’s *r* = 0.9557, *n* = 24, α = 0.05, two-tailed), the achieved statistical power exceeded 99%. Even for the lowest reported correlation, such as between central WF-OCTA VD-ISI and UWF-FA ISI (*r* = 0.7783), the power remained above 99%, indicating sufficient sensitivity to detect associations of this magnitude in the available sample. However, subgroup analyses examining agreement patterns across ischemia severity levels – particularly in eyes with severe ischemia – were limited by small sample sizes, restricting statistical power and the robustness of stratified inferences. Accordingly, while our observations demonstrate proportional field-dependent underestimation with increasing ischemia, larger cohorts are required to confirm this pattern before firm clinical recommendations rather than explorative suggestions can be made.

## Discussion

This study provides the first quantitative head-to-head comparison between the newly introduced DREAM WF-OCTA system and UWF-FA, showing that advanced WF-OCTA technology enables reliable non-invasive ischemia assessment with strong correlations to UWF-FA while revealing persistent field-of-view limitations in more pronounced nonperfusion. Its novelty lies in two key aspects: (i) to the best of our knowledge, it represents the first quantitative comparison of DREAM WF-OCTA with UWF-FA, leveraging the device’s increased field of view compared to previous OCTA platforms, and (ii) it validates the previously published VMseg algorithm on DREAM datasets, demonstrating cross-platform applicability for semi-automated ischemic index analysis. Our results further indicate that agreement varies with ischemia severity due to proportional bias: central WF-OCTA provides reliable estimates in mild ischemia (ISI ≤ 20%), montage WF-OCTA extends this applicability to moderate ischemia (ISI ≤ 40%), but both protocols increasingly underestimate ischemic burden in severe cases.

The DREAM OCT system addresses recognized limitations of previous WF-OCTA devices and studies [17–22] by enabling single-scan acquisition of 130° coverage, substantially exceeding earlier systems such as the Zeiss Plex® Elite 9000 (15 × 15 mm) or Canon Xephilio OCT-S1 (23 × 20 mm) [6, 23]. Our validation of previously developed VMseg on DREAM data (Dice-Sørensen coefficient 0.72 ± 0.12) confirms reliable semi-automated segmentation comparable to the Zeiss platform [10]. Unlike proprietary software solutions restricted to specific hardware platforms, VMseg provides transparent, reproducible analysis that facilitates cross-modality comparisons, making it particularly suitable for research applications such as our comparative study.

Notably, the regression slopes comparing WF-OCTA with UWF-FA were consistently below 1 (0.5364 for central and 0.8432 for montage), indicating proportional underestimation with increasing nonperfusion. The crossover with the identity line (y = x) occurs at approximately 14% (central) and 49% (montage) ISI, beyond which WF-OCTA increasingly underestimates ischemia. Although the correlation remains high, the absolute difference between modalities grows with ischemia severity: a pattern reflected in the Bland-Altman analysis, which demonstrated progressively widening bias at higher ISI values. These findings confirm that field-of-view constraints are not eliminated even with montage OCTA and become increasingly relevant in advanced disease. Conversely, in mild ischemia, OCTA may overestimate ISI, potentially due to improved detection of central capillary dropout or misclassification of peripheral signal attenuation as nonperfusion.

**Fig. 5:**
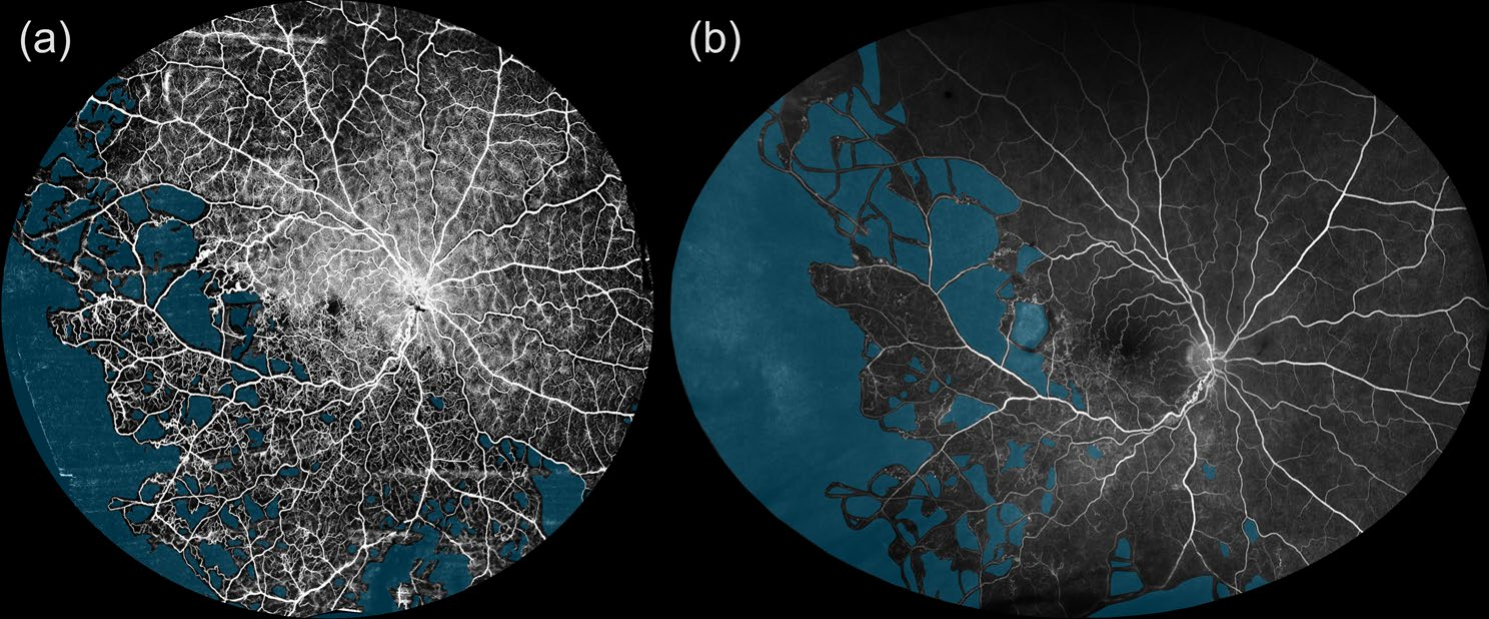
Comparative visualization of retinal ischemia using WF-OCTA and UWF-FA. (**a**) Montage WF-OCTA image acquired with DREAM OCT™, showing detailed microvascular structures. Zones of retinal nonperfusion are marked in blue. (**b**) Corresponding UWF-FA image of the same eye. While it provides broader peripheral coverage, the level of detail in capillary structures is reduced compared to WF-OCTA

Our Bland-Altman analysis further reinforces this insight: while correlation between WF-OCTA and UWF-FA remains consistently strong, absolute measurement agreement varies systematically with ischemia severity. In mild to moderate ischemia (ISI ≤40%), montage WF-OCTA demonstrates good absolute agreement with UWF-FA. However, the proportional bias becomes pronounced in severe ischemia, as peripheral nonperfusion extends beyond even the expanded DREAM field of view. This limitation is illustrated in Fig. 5, where far-peripheral dropout is clearly visible on UWF-FA but missed by montage WF-OCTA.

Recent evidence suggests that clinically significant nonperfusion in diabetic retinopathy predominantly occurs in the mid-periphery [16], which falls within the DREAM system’s coverage. Therefore, the far-peripheral detection limitations may have reduced clinical impact for routine monitoring, while UWF-FA remains essential for comprehensive assessment in advanced disease stages.

Our findings align with recent literature while revealing important differences from previous comparative studies. In a case report, Kim et al. highlighted DREAM OCT’s potential in diabetic retinopathy management, emphasizing its superior clarity in delineating nonperfusion regions [24]. However, our quantitative analysis demonstrates that even this advanced system underestimates ischemic burden in severe retinal nonperfusion. This systematic underestimation in severe ischemia represents a consistent finding across OCTA platforms, suggesting that field-of-view limitations persist despite significant technological advances. Studies using different OCTA field sizes have reported varying correlations with UWF-FA parameters: Decker et al. showed correlations of only 0.482 using 3 × 3 mm OCTA scans [20], while Glacet-Bernard et al. demonstrated significant correlations of 0.618 between vessel density and UWF-FA ischemic index using 12 × 12 mm OCTA montages [17]. Our study’s substantially stronger correlations suggest that the DREAM system’s expanded coverage represents a significant improvement over both smaller field systems and earlier approaches, yet the fundamental limitation in severe peripheral ischemia persists. Sawada et al. demonstrated that OCTA has higher detection rates for nonperfusion areas within its scan range but is restricted by the limited field-of-view for peripheral pathology [5].

Unlike proprietary software solutions restricted to specific hardware platforms, VMseg provides transparent, device-independent analysis that facilitates cross-modality comparisons and research applications. This represents a significant advancement over previous studies that relied on qualitative assessments [17, 18] or time-intensive manual segmentation prone to interobserver variability [19–22]. Our approach enables standardized ischemia quantification across different imaging platforms, supporting broader clinical adoption and research consistency. The algorithm’s performance on DREAM data validates its applicability beyond the original Zeiss platform, addressing a key limitation in current WF-OCTA research.

Based on our findings, we suggest a tiered imaging approach in which WF-OCTA, as implemented in the DREAM system, serves as a non-invasive first-line tool for screening and monitoring early to moderate retinal ischemia. It is particularly suitable for patients requiring frequent follow-up or in whom fluorescein angiography is contraindicated.

Despite improved coverage, WF-OCTA remains limited in detecting far peripheral pathology. In cases of suspected severe ischemia or when comprehensive vascular assessment is needed, including disease activity in exudative retinopathies, such as familial exudative vitreoretinopathy (FEVR) or Coats’ disease, UWF-FA remains essential. The DREAM system expands non-invasive applicability but does not replace dye-based imaging.

Our study has several limitations. The sample size (24 eyes) constrains subgroup analyses by disease type and severity. Disease-specific regional patterns in diabetic retinopathy versus retinal vein occlusion require validation in larger cohorts. Additionally, our single-center design limits generalizability across different populations and imaging protocols.

Future studies should focus on larger multi-center validation studies with disease-specific regional divided subgroup analyses, longitudinal assessment of DREAM’s utility in monitoring disease progression, and development of hybrid imaging protocols that optimize the complementary strengths of both modalities.

## Conclusion

This study presents the first quantitative comparison between the DREAM OCT system and UWF-FA, demonstrating that next-generation WF-OCTA enables reliable non-invasive ischemia assessment with strong correlations to UWF-FA. However, accuracy remains highly dependent on field of view and extent of nonperfusion area, with proportional underestimation increasing with nonperfusion severity, as shown by Bland-Altman analysis. Central WF-OCTA offers limited peripheral assessment, while montage WF-OCTA provides improved but incomplete agreement with UWF-FA. The integration of device-independent semi-automated segmentation enhances reproducibility and supports broader clinical adoption. WF-OCTA should be considered complementary to UWF-FA rather than a replacement, particularly in cases with severe ischemia. Future field-of-view expansion and algorithm refinement could further enhance clinical utility while UWF-FA remains essential for comprehensive peripheral assessment in high-risk patients.

## Data Availability

The datasets used and/or analyzed during the current study are available from the corresponding author upon reasonable request.

## Abbreviations

OCTA: Optical Coherence Tomography Angiography
UWF-FA: Ultrawide-Field Fluorescein Angiography
WF-OCTA: Wide-Field Optical Coherence Tomography Angiography
ISI: Ischemic Index
VD: Vessel Density
VD-ISI: Vessel Density-Derived Ischemic Index
VMseg: Variance-Map-Based Segmentation Algorithm
